# Study of Plugging Compositions Based on Synthetic Resins for Repair and Insulation Work in Wells

**DOI:** 10.3390/polym16142077

**Published:** 2024-07-21

**Authors:** Svetlana V. Aksenova, Lyubov A. Magadova, Sergey I. Kudryashov, Mikhail A. Silin, Aleksandr N. Kulikov, Artem V. Gevorkian, Denis D. Polyakov

**Affiliations:** 1 Department of Technology of Chemical Substances for the Oil and Gas Industry, Gubkin University, 119991 Moscow, Russia; assist502@nestro.ru (S.I.K.); silin.m@gubkin.ru (M.A.S.); ank-_1@mail.ru (A.N.K.); gevorkyan2001@bk.ru (A.V.G.); denispolyakov916@gmail.com (D.D.P.); 2 World-Class Research Center «Efficient Development of the Global Liquid Hydrocarbon Reserves», National University of Oil and Gas (Gubkin University), 119991 Moscow, Russia; 3 Zarubezhneft JSC, 101000 Moscow, Russia

**Keywords:** repair and insulation works, plugging composition, polymer, phenol-formaldehyde resin, hardener

## Abstract

This research is aimed at analyzing existing plugging compositions and developing a new plugging composition based on phenol-formaldehyde resin. This paper presents the results of studies of a hardening composition based on phenol-formaldehyde resin for repair and insulation work in wells. The plugging composition consists of two parts: Component “A” (resin and additives) and Component “B” (hardener). A resol-type water-soluble phenol-formaldehyde resin was selected for testing. The resin was additionally modified with special additives to improve performance properties. A mixture of acids was chosen as a hardener. Concentrations of resin and hardener were selected to ensure optimal loss of fluidity of the composition for different temperatures. The main physicochemical properties of the plugging composition were determined. The elastic-strength characteristics of the developed composition after curing at various temperatures (Poisson’s ratio, Young’s modulus, and compressive strength) were assessed. It has been experimentally proven that samples based on phenol-formaldehyde resin do not collapse completely under load but undergo longitudinal and transverse deformations. As the amount of hardener in the system increases, the compressive strength decreases. The presence of the elastic-strength properties of cementing compositions based on synthetic resins distinguishes them favorably from hardening compositions based on cement and microcement.

## 1. Introduction

Today, due to high water cut levels, a large amount of production wells are not being exploited. The main technical reasons for water flooding include leaks and defects in the production casing, violation of the tightness of the column space, interlayer flows, and the flow of bottom water to the bottomhole, as well as the formation or injection of water through washed-out highly permeable zones. The occurrence of leaks in production strings can be associated both with the quality of the primary cementing and the operating conditions of the wells themselves. Repair and insulation work (RIW) plays a key role in restoring integrity and eliminating threats to the continuous operation of wells.

Currently, all insulating materials for repair and insulation work in wells can be divided into five large groups according to their mechanism of action [1]:

1. Hardening compositions;
2. Gel-forming compositions;
3. Foam and emulsion compositions;
4. Sediment-forming materials;
5. Combined materials.

It is worthwhile dwelling in more detail on hardening compositions for RIW. Traditionally, Portland cement was used as the main material for RIW as the most affordable and durable material. Well cement is a dispersed system consisting of a mixing liquid (mainly water) and a mineral binder. During the process of hydration, the cement mortar hardens and forms a durable stone.

From the point of view of technical requirements, plugging compositions must have optimal fluidity for ease of injection while ensuring rapid thickening and strength gain after completion of the process. Their ability to penetrate the smallest pores and microcracks while, at the same time, being able to prevent spreading in large cracks under the influence of their weight is critical. Also important are sedimentation resistance, adhesion to casing pipes, and adhesion to rocks, as well as resistance to groundwater and the stability of properties when changing temperature and pressure. An important criterion for the applicability of a plugging composition is the penetrating ability of the solution. Obviously, for deeper penetration of the plugging composition into the pores of the formation, its reagents must have the smallest diameter of cement particles.

To date, formulations of hardening plugging compositions based on microcement [2], magnesium cement, and other structure formers with smaller particle sizes have been developed to improve penetrating ability. Such suspensions are used for work in which the use of traditional cement is impractical.

In cases where the maximum possible penetration of the insulating material is required, the use of cement mortars may be ineffective. Cement compositions have low filterability into small pore channels of formations. Therefore, it is advisable to use synthetic resins. Resin-based compositions, being ideal solutions, do not contain suspended solid particles in the solution. Such polymer cement compositions are capable of penetrating deeply into the porous medium of formations and, over time, forming a durable and load-resistant stone.

Phenol-formaldehyde resin-based plugging compositions are designed to restore the tightness of production strings, prevent leaks, and strengthen wells.

### 1.1. Grouting Compositions Based on Synthetic Resins

The production of synthetic resins is the result of the transformation of the original low-molecular compounds (LMCs; monomers) into high-molecular compounds (HMCs). This is possible using polymerization or polycondensation processes. 

All resins differ in chemical composition, rheological properties, solubility in water, and pH value. Hardened material is characterized by compressive strength, tensile strength, bending strength, and the degree of shrinkage of the resulting stone [3].

Of particular interest for laboratory research and practical application are phenol-formaldehyde and epoxy resins. Only phenol-formaldehyde resin was considered in our study.

Phenol-aldehyde polymers, which are the result of a polycondensation reaction between aldehydes and phenol, occupy a significant place in the chemical industry and materials science. These resins, also known as phenol-formaldehyde resins (PFRs), are a category of synthetic polymers with unique properties that allow them to be classified as reactoplasts or thermosets depending on their ability to be heat-treated and cured [4].

The process of condensation of phenols with aldehydes is influenced by various factors, among which the type of starting materials and reaction conditions are of key importance. This makes it possible to obtain two main types of phenol-formaldehyde resins: thermoplastic and thermosetting. Thermoplastic resins, which are often referred to in the industrial environment as novolacs, are capable of transitioning from a solid to a viscous-flow state and back under the influence of heat without changing their chemical structure. On the other hand, thermoset resins, or resoles, undergo chemical transformation when heated to form spatially cross-linked polymer networks, making them irreversibly hard and insoluble. It is thermosetting resins that are of interest for well operations as the basis for a waterproofing composition, so we will consider resole PFRs.

Additives used to create plugging material help regulate the setting speed and plastic properties of the cementing solution, as well as the degree of shrinkage of the hardened composition. Such additives are curing initiators and regulators, thickeners, and weighting/lightening additives, as well as solvents and thinners of various types.

The main additive that is studied in the scope of this work is the hardener. The literature presents many components that help to harden phenol-formaldehyde resin. For example, in the article [5], PFR is cured by high temperatures (130–200 °C) and the addition of benzene sulfonic acid or phosphoric acid, but this component has a big disadvantage because not all wells have a formation temperature equal to 200 °C. In the article [6], the authors suggest using ammonium sulfates or chlorides; the disadvantage of this method of curing can also be considered high curing temperatures of 90–100 °C, which in the case of most wells in Western Siberia, for example, is impossible [7]. In the patent [8], the authors propose to cure the resin using urotropine; this method has disadvantages because, in the process of crosslinking the resin, a large amount of water is released, which negatively affects the efficiency of the RIW. The patent [9] uses a mixture of boric anhydride and hegsamethylenetetramine to cure PFR. This method also has some limitations as the hardener components are expensive and not always available in the field. One of the most popular PFR curing agents, resorcinol [10], has several disadvantages, including the high cost of the curing agent, toxicity, and limited availability in oilfield operations.

Based on the above-mentioned, the development of an effective hardener is an important and urgent goal for the entire oilfield industry. The hardener should be economically accessible, non-toxic, and have the ability to control the curing time of the plugging compound within wide limits.

### 1.2. Advantages of Using Synthetic Resins over Cement Compositions

As described earlier, in most cases, compositions based on mineral binders are used as materials for RIW. Inorganic substances, such as cement, clay, and gypsum, usually act as structure-forming agents. Such systems are suspensions. A number of works are devoted to comparing the properties of cement and resin [11,12,13,14,15,16].

The main focus is on assessing the penetrating ability of the compositions. In the article [12], the penetration ability of a composition based on Portland cement and epoxy resin into a pack of sand was compared under laboratory conditions. The authors used sand with particle sizes ranging from 50 to 250 microns and formed sand packs in plastic syringes. Cement mortar meeting API requirements was placed on top of the compacted pack. As the cement slurry lost liquid, a dark layer formed at the sand-cement interface over time. The liquid could pass through the sand pack, unlike the solid cement particles.

The same procedure was repeated using a particulate-free epoxy resin composition. Almost immediately, the liquid resin easily penetrated the sand pack and continued to flow in and out of the syringe tip. Over time, the resin composition formed a three-dimensional cross-linked structure and hardened throughout the sand.

When carrying out repair and insulation work, it is important to determine the well’s specific injectivity index. Well injectivity is a characteristic of an injection well showing the possibility of injecting a working agent (water, gas, steam, etc.) into the formation, determined by the volume of mixture injected into the formation per unit time. 

The well’s injectivity depends on the repression created at the bottom of the well (the difference between the bottomhole and reservoir pressures), the perfection of formation penetration, and its thickness and permeability for the injected fluid. 

In technological calculations, the well injectivity index is also used, equal to the ratio of the amount of working agent injected into the formation per unit of time to the repression created at the bottom of the well during injection.

The injectivity index [m^3^/day/MPa)] of an injection well is determined by Equation (1):

(1) $$K^{\prime }=\frac{Q_{f}}{\left(P_{bot}-P_{res}\right)},$$

where *Q_f_* is the fluid flow rate pumped into the well (m^3^/day); and *P_bot_−P_res_* is the repression created at the bottom of the well (difference between bottomhole and reservoir pressures) (MPa).

Before carrying out work, it is necessary to conduct an injectivity test by pumping a liquid that does not contain solid particles through the annulus and calculating the injectivity coefficient.

In [11,13], the possibility of using different types of plugging materials depending on the well’s injectivity was studied. The injectivity index [psi*min/barrel] of the well was calculated as follows (Equation (2)):

(2) $$K=\frac{P_{dis}}{Q},$$

where *P_dis_* is the discharge pressure (psi); and *Q* is the volume of pumped liquid (barrel/min). 

There is an inverse relationship between injectability and the injectivity index. 

As shown in Table 1 [11], as the injectivity index increases, a finer particle size is required. 

The need for polymer resin-based systems increases as the well injectivity decreases. 

Thus, resin-based compositions, due to the absence of solid suspended particles in the composition, can penetrate deeply into formations and ensure complete sealing of microcracks and channels both in the formations and the cement. 

In addition to penetrating ability, scientists also studied other properties of resin-based compositions. In [15], cement and resin compositions were compared in terms of compressive and flexural strength, density, elastic modulus, and other properties (Table 2).

According to the authors, the resin-based system is superior to cement in its strength characteristics and has pronounced elastic properties. 

Resin-based compositions differ in the range of density (from 0.75 to 2.5 g/cm^3^) and dynamic viscosity (from 10 to 2000 mPa*s). An “aging” test of the material showed that, unlike cement, the mechanical properties do not decrease over time. 

The hardening process of the thermoset resin can be controlled by adding special reagents for specific formation temperatures. Varying the ratio of base (resin) and hardener helps to obtain the optimal thickening time for the successful injection of the composition into the well.

There is now an urgent need to reconsider outdated methodologies for plugging and abandoning oil and gas wells, as well as depleted reservoirs. Many previously abandoned wells are now flooded, for example, due to the destruction of cement. As a result, many professionals are looking to use alternative materials that are more durable and reliable. However, as is often the case in all industries, the introduction of new technologies is associated with economic difficulties.

Quite often, downhole tools and equipment are seriously damaged, i.e., pipes are destroyed, and valves or packers are damaged. As a result of these problems, pumping cement as the main plugging material may not be the optimal solution. In these cases, the use of synthetic resins can be a good alternative to achieve the required goal. The deterioration of cement can often lead to the formation of gaps and cracks. These include areas between the formation and the primary cement or between the primary cement and the casing, resulting from improper wellbore mounting and failure to remove drilling fluid from the wellbore. In addition, if hydrostatic pressure is not retained by the cement in the gas-bearing formation, annulus fluids may move into the wellbore before cement hardening, resulting in the formation of gas channels.

These problems can compromise the integrity of wells, often leading to remediation work that can add additional operating costs. The need for new materials has long been recognized by the industry, and research is currently being conducted to identify and test alternative materials for cementing and repair and insulation work. 

Tests carried out on a thermosetting resin in [16] showed that the material has the necessary mechanical properties to meet the stringent requirements for plugging materials. In the study, resin samples were exposed to crude oil, methane, CO_2_, and H_2_S.

Chemical exposure was carried out at intervals of 1, 3, 6, and 12 months on prism-shaped resin samples (10/10/80 mm). For permeability testing, 5 mm disc-shaped polymer prisms were used. Curable resins have been proven to have unique properties that allow them to withstand the harsh chemical conditions typically found in a well while maintaining integrity beyond the life of the well. The advantages of using thermoset resins are due to the physical characteristics of the polymers themselves.

Resin systems typically consist of a base polymer to which one or more additives are added to control setting time and, in some compositions, viscosity. Unlike cement, where the initiation of the cement hydration reaction and associated injection time begins with the addition of water, resin polymerization reactions are not possible unless thermodynamic and kinetic criteria are met. The hardening time of the resin depends on the temperature and structure of the polymer. A temperature that is much higher than the temperature for which the thermoset resin was designed will increase the kinetics of the polymerization reaction and reduce the specified setting time. Conversely, a temperature lower than necessary will inhibit the setting of the material.

Thus, the properties of hardened resins allow them to withstand the chemistry typically found in the well and maintain reliable integrity. Thanks to the ability to control the setting time, effective resin-based compositions help reduce economic costs during work and provide ease of operation.

Other notable properties are good adhesion to steel, compatibility with most fluids and cements, and exceptional chemical resistance. The thermosetting resin hardens into a durable and flexible material. The hardening process is influenced by the formation temperature and the reaction mechanism of the resin with the hardener, which allows you to accurately pump and adjust the thickening time in accordance with the requirements of the subsoil user for each well.

The strength of stone is another important indicator of hardening materials. The well space is subjected to static and dynamic alternating loads. Plugging materials based on resins have pronounced elastic properties, which distinguish them favorably from cement compositions [17,18,19,20]. 

Synthetic resins as a material for RIW have a number of useful technological properties: low viscosity and good filterability into the pores and channels of the formation, the absence of suspended particles, low shrinkage during hardening, high strength, and adhesion to the rock surface.

Thus, synthetic resins have a wide range of applications [13,21,22,23,24,25,26,27,28]:

1. Isolation of water inflow;
2. Eliminating leaks in the production casing;
3. Fastening the bottomhole zone of sand-producing wells;
4. Elimination of inter-casing pressure;
5. Additional strengthening of cement.

This paper presents the research results of the developed composition based on resol phenol-formaldehyde resin.

## 2. Materials and Methods

### 2.1. Materials

In the experimental part of the work, a water-soluble phenol-formaldehyde resin of the resol type was used with an amount of free formaldehyde no more than 4 wt% (SFZH-3027B, “Himsintez”, Chapaevsk, Russia). 

To improve the performance characteristics, functional additives were introduced into the resin: polyhydric alcohol (“Petrokhim”, Belgorod, Russia) and a sulfo derivative of lignin (“Solikamskbumprom”, Solikamsk, Russia). 

As a hardener for the phenol-formaldehyde resin, a mixture of acids with a corrosion inhibitor was selected, which makes it possible to regulate the time of loss of fluidity of the composition at different temperatures. The inhibitor concentration for the hardener was selected on the basis of steel corrosion rate studies using the gravimetric method. 

Thus, the plugging composition consists of two parts:

Component “A”:

- Resol phenol-formaldehyde resin—50.0–80.0% vol.;
- Polyatomic alcohols—50.0–20.0% vol.;
- Lignin sulfo derivative—5.0–25.0 wt% (over).

Component “B” (hardener):

- Mixture of mineral acids (concentration 10.0–30.0 wt%)—95.0 wt%;
- Corrosion inhibitor—5.0 wt%.

### 2.2. Experimental Methods

#### 2.2.1. Preparation of Grouting Solution

To prepare the grouting solution, the required mass of resin, previously modified with additives, was placed in a glass measuring cup and weighed on an analytical balance. The glass with resin was placed on the IKA EUROSTAR digital mixing device (IKA-Werke GmbH & Co. KG, Staufen, Germany). Next, the hardener was added. Stirring was carried out until the components were completely dissolved using a top-drive paddle mixer at a speed of 300–400 rpm. After mixing, freshly prepared solutions were poured into sealed containers with screw caps.

#### 2.2.2. Determination of the Loss of Fluidity Time of Plugging Composition

To determine the time of loss of fluidity of the composition, all samples were placed in a drying oven (LOIP LTD, Saint-Petersburg, Russia), preheated to the experimental temperature, and visually assessed every 15–30 min. The moment the composition began to thicken was recorded visually by the loss of fluidity of the composition. When the vessel with the plugging composition was tilted by 45 degrees, the meniscus did not shift.

#### 2.2.3. Determination of the Degree of Hardening of the Plugging Composition

The test was carried out using a Vicat needle (VNIR, Moscow, Russia). The needle of the device was brought into contact with the surface of the thickened plugging composition prepared according to Section 2.2.1. 

The measurement was based on the method of lowering a needle into the prepared grouting solution to capture the beginning and end of the hardening of the composition. The beginning of hardening can be considered the time from the preparation of the plugging mortar until the moment when the needle does not reach, by 2–4 mm, the plate on which the sample was installed. The end of hardening can be considered the time from the preparation of the plugging mortar until the moment when the needle was lowered by no more than 1–2 mm into the plugging composition.

#### 2.2.4. Determination of the Elastic-Strength Characteristics of the Plugging Composition

**Figure 1:**
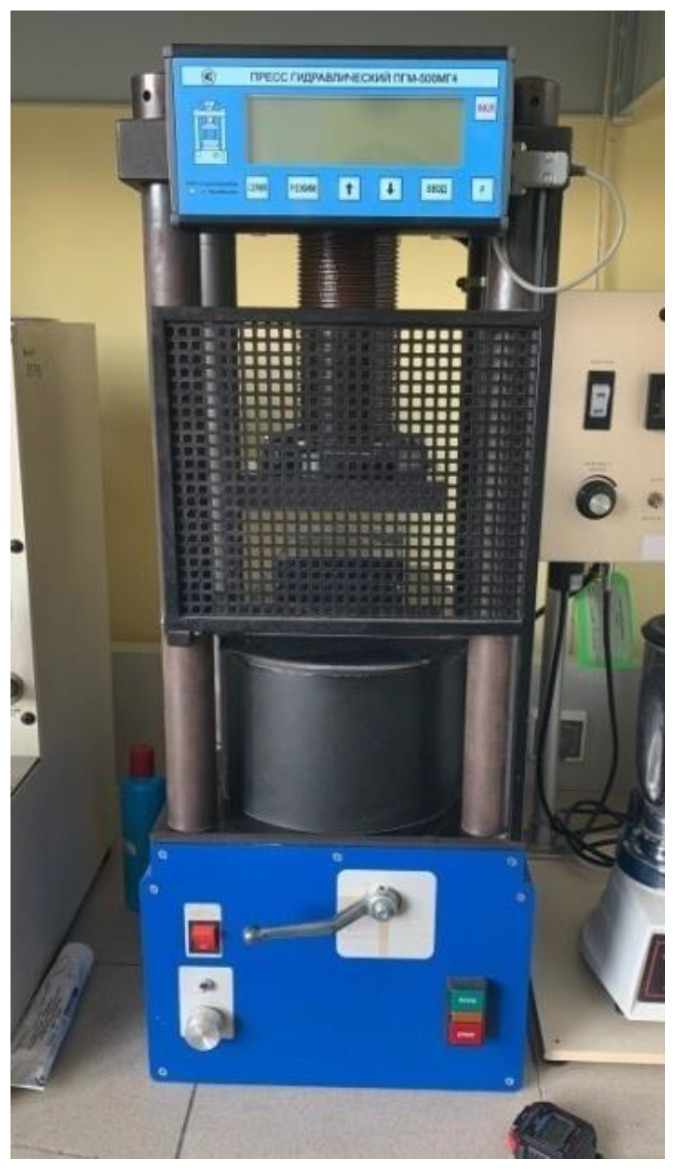
Hydraulic press PGM-500 MG4.

The elastic-strength properties of hardened resin-based stones were studied using a PGM-500 hydraulic press (SKB Strojpribor, Chelyabinsk, Russia). Compressive strength testing of the samples was carried out in accordance with instructions for the device (Figure 1). Cylindrical samples were measured before and after loading using a caliper. Then, the values of longitudinal and transverse strains were calculated, and Poisson’s ratio and Young’s modulus for the samples were determined.

For testing, cylindrical samples of a certain size were made. A sample of plugging stone was installed in a press between two plates through which compression was carried out. At least three samples were tested for compression. The compressive strength was taken to be the average of the two largest values, calculated with an accuracy of 0.1 MPa.

#### 2.2.5. Determination of the Freezing Point of the Components of the Composition

**Figure 2:**
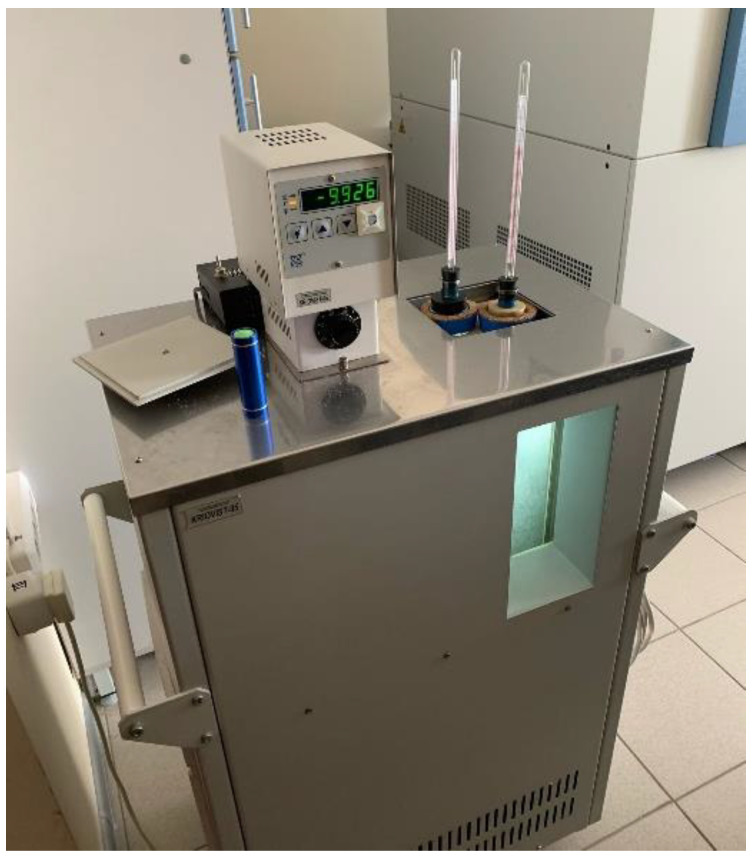
CRYO-VIS-T-05 cooling constant temperature bath.

The study was carried out in accordance with GOST [29]. A test tube with a certain amount (6 mL) of the test sample and a thermometer were placed in a special vessel with a larger volume. This structure was placed in the CRYO-VIS-T-05 (TERMEX, Tomsk, Russia) with a cooling mixture, the temperature of which was pre-set 5 °C lower than the temperature to determine the freezing point (Figure 2).

When the sample in the test tube reached the freezing temperature, the test tube was tilted at an angle of 45° and kept in this position for 1 min. After finding the solidification boundary (the transition from mobile to immobile or vice versa), the determination was repeated, with the test temperature decreasing or increasing by 2 °C, until a temperature was established at which the meniscus of the sample remained immobile and when the test was repeated at a temperature 2 °C higher, it shifted. This temperature was recorded as the set temperature for this experiment.

## 3. Results and Discussion

### 3.1. Selection of an Additive to Reduce the Freezing Point of the Phenol-Formaldehyde Resin

The original phenol-formaldehyde resin is a mobile transparent liquid of a light brown to dark cherry color, the freezing point of which is, as a rule, not lower than −15 °C. Sometimes, repair and insulation work must be carried out at low temperatures. Therefore, there is a need to reduce the freezing point of the resin. 

Since this resin is a hydrophilic liquid, water-soluble mono- and polyhydric alcohols have been studied as reagents for reducing the freezing point. Some alcohols, such as methyl alcohol, effectively lowered the freezing point. However, they caused severe shrinkage of the hardened composition (more than 10%), which was an unacceptable result. Polyhydric alcohols showed the best effect. They caused virtually no shrinkage and helped to significantly reduce the freezing point. 

**Figure 3:**
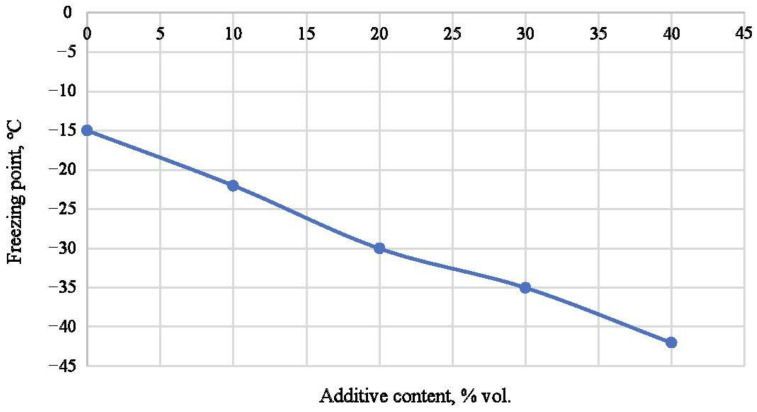
Dependence of the freezing temperature of the resin on the concentration of the modifying additive.

This study found the optimal ratio of resin and modifier to ensure a lower freezing point (Figure 3).

The graph shows that an increase in the content of the modifying additive leads to a decrease in the freezing temperature of the resin. However, an additive content of more than 40% reduces the quality of the hardened composition (including strength characteristics), so a further increase in the additive concentration is undesirable. 

### 3.2. Selection of an Additive to Reduce Water Separation

**Figure 4:**
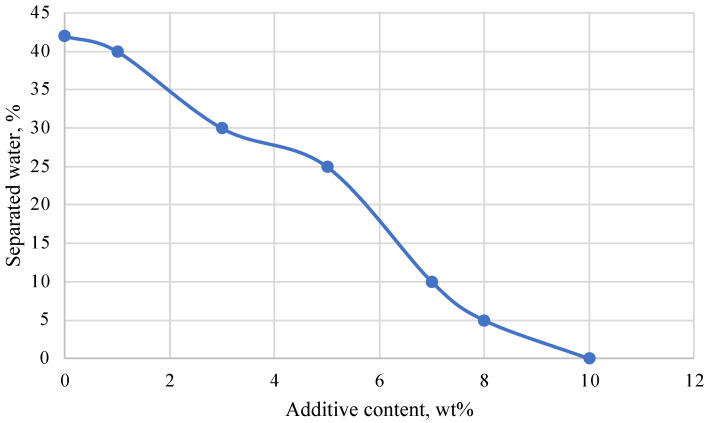
Dependence of the volume of separated water on the concentration of the additive.

During the hardening process of the plugging composition, water may be released from the resin after curing. To solve this problem, additives based on lignin sulfo derivatives have been investigated. It has been found that increasing the amount of additive in the resin reduces the volume of water released during hardening. The results of selecting the concentration of the additive are presented in Figure 4. As an example, a composition with the same ratio of resin and hardener was chosen. The samples were kept at 45 °C for 24 h. The graph shows that by increasing the additive concentration up to 10 wt%, the volume of separated water decreases to 0%.

### 3.3. Selection of Hardener Concentration

Resol resins are also capable of hardening during long-term storage, even at room temperature. During hardening, all water is separated from the polymer since as the molecular weight of phenol-formaldehyde resin increases, its solubility in water drops to zero. Depending on the hardening conditions, water separation can reach up to 60% of the resin weight.

Based on the literature data, studies have been carried out to assess the possibility of curing phenol-formaldehyde resin with hardeners of various chemical natures. 

Analysis of literature data showed that lowering the pH of the resin solution and adding diatomic phenol to phenol-formaldehyde resins accelerates the process of gelation and stone formation [6,10,30,31,32,33].

To harden most phenol-formaldehyde resins, acidic hardeners (mineral and organic acids) or their acid salts (aluminum chloride, aluminum sulfate, and ferric chloride) are required. A common disadvantage of acid-hardening plugging compounds is their high corrosion ability toward rock, cement, and casing metal. Therefore, a corrosion inhibitor is selected for such hardeners. When heated, the hardening of resol resins is accelerated by the addition of the alkaline earth metal oxides CaO, MgO, and BaO. 

**Figure 5:**
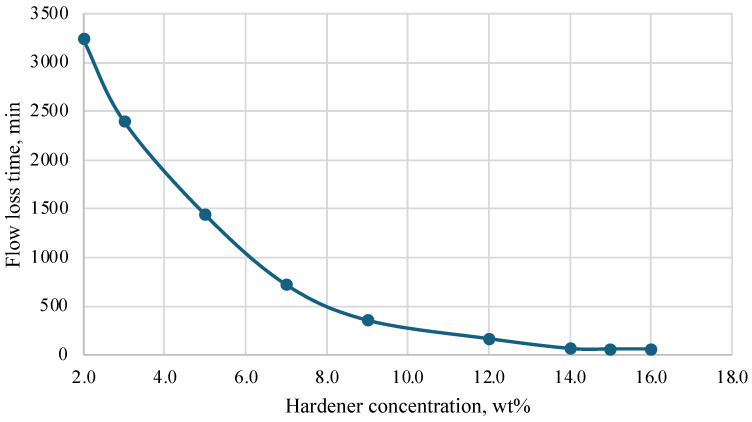
Dependence of flow loss time on hardener concentration (25 °C).

**Figure 6:**
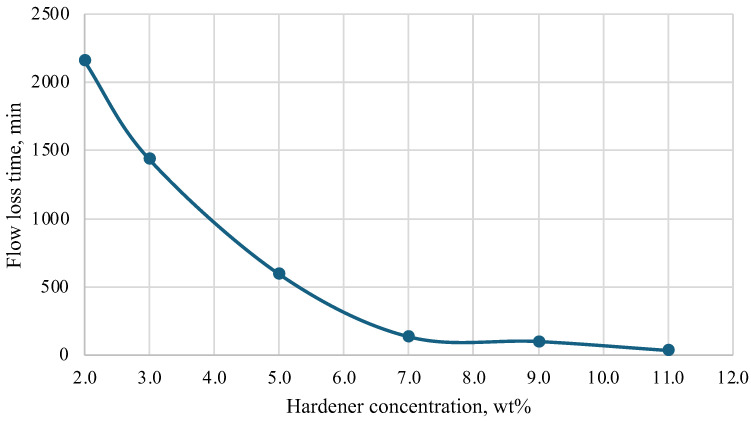
Dependence of flow loss time on hardener concentration (40 °C).

**Figure 7:**
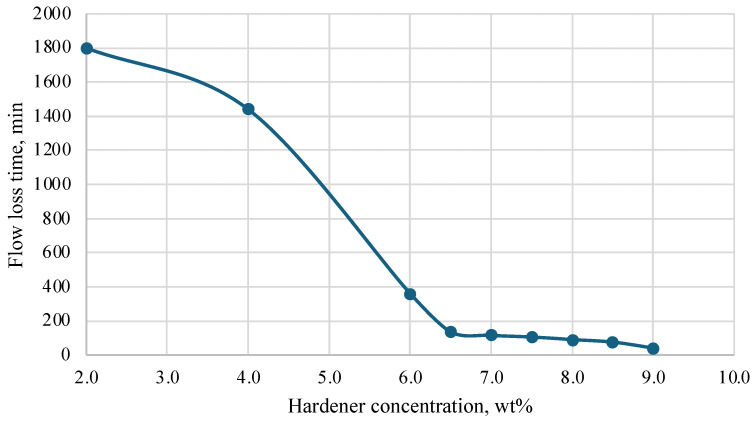
Dependence of flow loss time on hardener concentration (60 °C).

**Figure 8:**
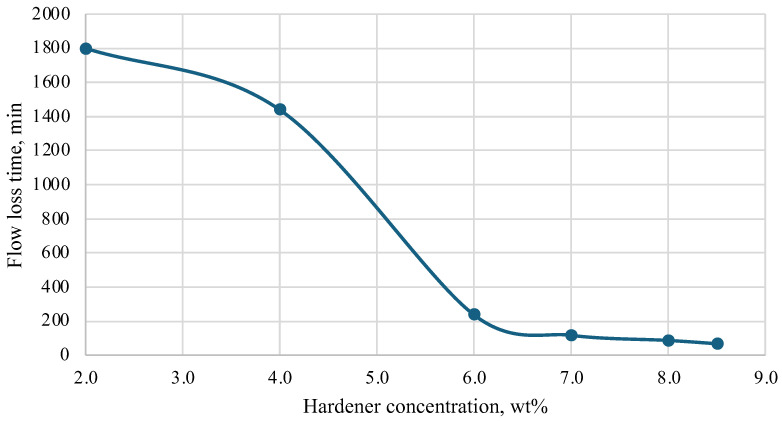
Dependence of flow loss time on hardener concentration (70 °C).

During the research, a hardener was selected based on a mixture of acids and a corrosion inhibitor. Initial laboratory tests were conducted at temperatures of 25 °C, 40 °C, 60 °C, and 70 °C. Based on the results obtained, a graph was constructed that helps to select the concentration of the hardener depending on the required thickening time of the plugging composition. The results are presented in Figure 5, Figure 6, Figure 7 and Figure 8.

Depending on the technology used at the well, the time of loss of fluidity of the plugging composition should be in the range of 2 to 10 h, and complete hardening should be within 24 h. It should be noted that both excess and deficiency of hardener worsen the characteristics of the plugging composition. An insufficient amount of hardener in the composition leads to a significant slowdown in the thickening process and further strength gain. In some cases, a severe lack of hardener leads to the fact that the composition does not lose fluidity even after 24 h, and stone formation does not occur. An excess of hardener, on the contrary, greatly accelerates the loss of fluidity and hardening of the compositions and leads to the formation of cracks in the hardened samples and shrinkage in volume.

As a result of the study, the most optimal values of hardener concentration were selected for different temperatures, taking into account the time of injection and filtration of the composition into the well space. 

### 3.4. Evaluation of the Elastic-Strength Properties of the Developed Composition

**Figure 9:**
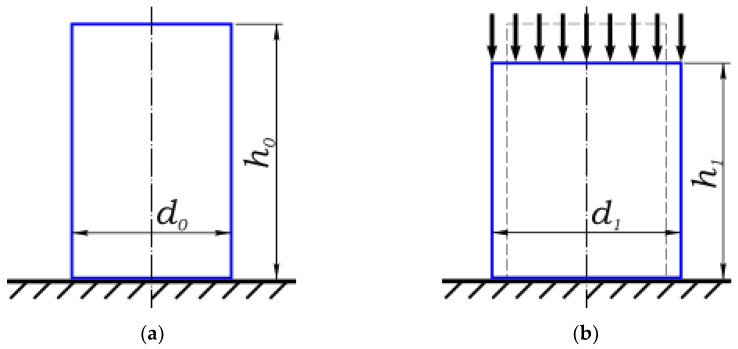
(**a**) Cylindrical sample before loading; (**b**) Cylindrical sample after loading.

Schematically, the process of compression of a cylindrical sample under a press is represented in Figure 9 and Equations (3) and (4). In (a), h_0_ is the initial longitudinal size; and d_0_ is the initial transverse size (in this case, diameter). In (b), h_1_ is the final longitudinal size; and d_1_ is the final transverse size (in this case, diameter).

*h*_1_ = *h*_0_ − ∆*h* (3)

*d*_1_ = *d*_0_ + ∆*d* (4)

where Δ*h* and Δ*d* are the absolute longitudinal and transverse strains, respectively.

#### 3.4.1. Determination of Poisson’s Ratio

Poisson’s ratio (υ) shows the relationship between the longitudinal and transverse deformations of an element and characterizes the elastic properties of the material, depending on the nature of the material. The values are taken modulo since longitudinal (*ε_l_*) and transverse deformations (*ε_t_*) always have opposite signs. 

The ratio of absolute deformations to the corresponding initial sizes will show the relative deformations ε (Equations (5) and (6)):

(5) $$\varepsilon_{t}=\frac{∆d}{d_{0}},$$

(6) $$\varepsilon_{l}=\frac{∆h}{h_{0}},$$

The ratio of transverse and longitudinal deformations, in turn, determines the Poisson’s ratio of the cylinder material (Equation (7)).

(7) $$\upsilon =\left|\frac{\varepsilon_{t}}{\varepsilon_{l}}\right|$$

#### 3.4.2. Determination of Young’s Modulus (Modulus of Elasticity)

The elastic modulus is a physical quantity that characterizes the properties of a material to resist tension/compression during elastic deformation or the property of an object to deform along an axis when exposed to a force along this axis, showing the degree of hardness of the material. 

Young’s modulus (*E*) is calculated using Equation (8):

(8) Ε=FSχl=F·lS·χ

where *E* is the modulus of elasticity; *F* is force; *S* is the surface area over which the force is distributed; *l* is the length of the deformable rod; and *x* is the modulus of change in the length of the rod as a result of elastic deformation.

The compressive strength of plugging stones was studied using a PGM-500 hydraulic press. Tests were carried out on cylindrical samples. The test results are presented in Table 3, Table 4, Table 5 and Table 6. Samples with a flow loss time greater than 24 h (1440 min) were not tested. 

Based on the data in Table 3, Table 4, Table 5 and Table 6, it can be seen that at a temperature of 40 °C, the values of the compressive strength, as well as the elastic modulus of the samples are generally higher than those of the samples hardening at 25 °C. 

With an increase in the hardening temperature of the compositions, Young’s modulus increases in all samples.

It is known that the higher the value of Poisson’s ratio, the more elastic the material; the less, the more fragile the material. When the Poisson’s ratio is ν = 0, the material is absolutely fragile; when the Poisson’s ratio is ν = 0.5, it is absolutely elastic (rubber).

For samples hardening at 25 °C, Poisson’s ratio is much lower than for other plugging samples. The fragility of the samples was proven both by the calculations and the presence of samples that were destroyed during compression in a series of experiments.

**Figure 10:**
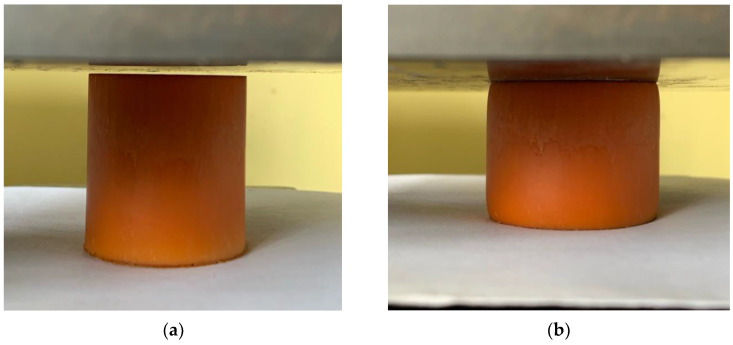
(**a**) Sample composition before loading; (**b**) Sample composition during compression.

Nevertheless, most of the samples did not collapse after loading. However, after removing the load, all samples did not completely return to their original dimensions; irreversible deformation occurred in longitudinal and transverse directions (Figure 10).

A comparison of the properties of samples based on phenol-formaldehyde resin with other materials is presented in Table 7.

In the process of perforation, acid treatments, hydraulic fracturing, and other operations, leaks in the annulus and damage to the well support may occur. Plugging materials based on mineral binders (for example, cement) have a rigid crystal lattice and do not have such pronounced deformation properties. Resins, exhibiting elastic properties, are able to withstand static and dynamic alternating loads.

As a result of research, we analyzed the compressive strength, Young’s modulus, and Poisson’s ratio for different materials used as plugging compositions. Compositions based on PFR and the presented hardener showed the best results in all three parameters, which indicates the effectiveness of the developed composition for well application.

## 4. Conclusions

This paper presents the results of studies of a hardening composition based on phenol-formaldehyde resin for repair and insulation work in wells. A resol-type water-soluble phenol-formaldehyde resin was selected for testing. To cure the resin, a hardener was selected based on a mixture of acids and a corrosion inhibitor.

The addition of polyhydric alcohols (no more than 40%) made it possible to reduce the freezing point of the original resin, which makes it easier to carry out work in winter. The addition of sulfo-derivative lignin to the resin helps reduce the amount of water separated from the resin during the curing process and minimizes it. Concentrations of resin and hardener were selected to ensure the optimal loss of fluidity of the composition at different temperatures. As the temperature increases, the rate of flow loss of the plugging solution also increases, so with increasing temperature, it is necessary to add less hardener to achieve optimal values (flow loss time is from 120 to 600 min, and time of full curing is within 24 h). The elastic-strength characteristics of the developed composition after curing at 25 °C, 40 °C, 60 °C, and 70 °C (Poisson’s ratio, Young’s modulus, and compressive strength) were assessed. The samples based on phenol-formaldehyde resin do not collapse completely under loads but undergo longitudinal and transverse deformations. The presence of the elastic-strength properties of plugging compositions based on synthetic resins distinguishes them favorably from hardening compositions based on different types of cement. So, the advantages of the developed composition are the low freezing point of components, no solid suspended particles in the composition, controllable curing time in a wide temperature range, and highly expressed elastic-strength properties.

## Figures and Tables

**Table 1:** Recommended type of injected material depending on the injectivity index [11].

Injectivity	The Injectivity Index	Type of Plugging Material
High	<2000	Normal API cement
	2000–4000	API Cement is a fine blend of 50–80% Class C, G, or H cement with 20–50% microcement
	4000–6000	Solid-free materials (resins/monomer) and microcement
Low	>6000	Solid-free materials (resins/monomer) Water-based monomer mixture

**Table 2:** Comparison of indicators of traditional cement and resin [15].

Property	Thermal-Activated Resin	Traditional Cement
Water permeability, mD	<0.5	1600
Compressive strength, MPa	77	58
Flexural strength, MPa	43	10
Failure flexural strain, %	1.9	0.32
E-modulus, MPa	2240	3700
Tensile strength, MPa	60	1
Density, S.G.	0.75–2.5	1.5+

**Table 3:** Determination of elastic properties of plugging stone (curing at 25 °C).

No.	Hardener Concentration, wt%	Flow Loss Time, min	h_0_, mm	d_0_, mm	h_1_, mm	d_1_, mm	Δh, mm	Δd, mm	ℇ_t_	ℇ_l_	υ	S, mm^2^	R, MPa	E, MPa
1	9.0	355	28.55	49.05	25.32	49.54	−7.73	0.49	0.010	−0.27	0.037	1888.63	8.88	32.81
2	12.0	164	28.46	49.6	26.33	49.93	−7.43	0.33	0.007	−0.26	0.025	1931.22	7.79	29.81
3	14.0	65	31.12	49.76	destruction of the sample	destruction of the sample	–	–	–	–	–	1943.70	7.88	–
4	15.0	80	28.83	49.9	27.45	49.63	−7.71	−0.27	−0.005	−0.27	0.020	1954.65	6.42	23.99
5	16.0	60	31.27	49.52	destruction of the sample	destruction of the sample	–	–	–	–	–	1925.00	5.22	–

**Table 4:** Determination of elastic properties of plugging stone (curing at 40 °C).

No.	Hardener Concentration, wt%	Flow Loss Time, min	h_0_, mm	d_0_, mm	h_1_, mm	d_1_, mm	Δh, mm	Δd, mm	ℇ_t_	ℇ_l_	υ	S, mm^2^	R, MPa	E, MPa
1	3.0	>1440	0	0	0	0	0	0	0	0	0	0	0	0
2	5.0	600	27.82	48.7	24.43	51.98	−8.36	3.28	0.07	−0.30	0.224	1861.77	13.06	40.13
3	7.0	140	28.25	48.84	24.43	51.98	−8.29	3.14	0.06	−0.29	0.219	1872.49	12.21	45.01
4	9.0	105	28.34	48.32	26.31	48.7	−6.62	0.38	0.008	−0.23	0.034	1832.83	10.93	46.78
5	11.0	40	29.63	48.76	destruction of the sample	destruction of the sample	–	–	–	–	–	1866.36	8.95	–

**Table 5:** Determination of elastic properties of plugging stone (curing at 60 °C).

No.	Hardener Concentration, wt%	Flow Loss Time, min	h_0_, mm	d_0_, mm	h_1_, mm	d_1_, mm	Δh, mm	Δd, mm	ℇ_t_	ℇ_l_	*v*	S, mm^2^	R, MPa	E, MPa
1	2.0	1800	0	0	0	0	0	0	0	0	0	0	0	0
2	4.0	1440	0	0	0	0	0	0	0	0	0	0	0	0
3	6.0	360	29.33	35.55	28.36	36.28	−3.46	0.73	0.02	−0.12	0.174	992.09	9.61	81.43
4	6.5	135	31.6	35.77	24.63	36.76	−3.37	0.99	0.03	−0.11	0.260	1004.40	11.63	109.04
5	7.0	115	32.64	34.79	25.7	35.9	−4.75	1.11	0.03	−0.15	0.219	950.12	14.62	100.46
6	7.5	105	30.72	35.74	29.23	36.17	−4.34	0.43	0.01	−0.14	0.085	1002.72	14.11	99.89
7	8.0	90	27.81	35.6	26.56	36.91	−4.55	1.31	0.04	−0.16	0.225	994.88	13.92	85.09
8	8.5	75	28.14	35.68	27.94	35.97	−4.21	0.29	0.01	−0.15	0.054	999.35	15.01	100.33

**Table 6:** Determination of elastic properties of plugging stone (curing at 70 °C).

No.	Hardener Concentration, wt%	Flow Loss Time, min	h_0_, mm	d_0_, mm	h_1_, mm	d_1_, mm	Δh, mm	Δd, mm	ℇ_t_	ℇ_l_	υ	S, mm^2^	R, MPa	E, MPa
1	2.0	1800	0	0	0	0	0	0	0	0	0	0	0	0
2	4.0	1440	0	0	0	0	0	0	0	0	0	0	0	0
3	6.0	240	28.02	35.61	27.06	36.32	−4.43	0.71	0.02	−0.16	0.126	995.44	17.78	112.47
4	7.0	120	27.94	35.6	27.17	36.69	−4.69	1.09	0.03	−0.17	0.182	994.88	15.40	91.74
5	8.0	90	24.93	35.58	destruction of the sample	destruction of the sample	–	–	–	–	–	993.76	19.22	–

**Table 7:** Elastic-strength properties of insulating compositions based on resins and other materials.

Material	Ultimate Compressive Strength, MPa	Young’s Modulus, MPa	Poisson’s Ratio
Glass **	-	0.56·105	0.25
Concrete **	10.0	(0.146–0.196)·105	0.16–0.18
Phenol-formaldehyde resin, 40 °C ***	12.06	40.12	0.22
Phenol-formaldehyde resin, 25 °C ***	8.88	32.81	0.04
Epoxy resin, 50 °C ***	- *	4.02	0.27
Rubber **	-	7.0	0.47–0.50

* When measuring the tensile strength on a hydraulic press, the sample did not collapse (peak value of load per unit area); ** Reference data [34]; *** Hardening temperature. Young’s modulus [MPa] is a physical quantity that characterizes the property of a material to resist tension or compression during elastic deformation and shows the degree of rigidity of the material. The higher the Young’s modulus, the less the deformation and the greater the material’s resistance to deformation. Poisson’s ratio is a physical quantity that determines the relationship between longitudinal and transverse deformations of a material and characterizes the elastic properties of the material.

## Data Availability

The data presented in this study are openly available in the article.

## Abbreviations and Symbols

RIW	Repair and insulation work
PFR	Phenol-formaldehyde resin
LMC	Low-molecular compound
HMC	High-molecular compound
h_0_	Initial longitudinal size
d_0_	Initial transverse size
h_1_	Final longitudinal size
d_1_	Final transverse size
Δh	Absolute longitudinal strains
Δd	Absolute transverse strains
ε_l_	Relative longitudinal deformations
ε_t_	Relative transverse deformations
υ	Poisson’s ratio
E	Young’s modulus, MPa
R	Ultimate compressive strength, MPa
